# Optimizing the Better Nights, Better Days for Children with Neurodevelopmental Disorders program for large scale implementation

**DOI:** 10.3389/frsle.2023.1158983

**Published:** 2023-05-04

**Authors:** Alzena Ilie, Matt Orr, Shelly Weiss, Isabel M. Smith, Graham J. Reid, Ana Hanlon-Dearman, Cary Brown, Evelyn Constantin, Roger Godbout, Sarah Shea, Osman S. Ipsiroglu, Penny V. Corkum

**Affiliations:** ^1^Department of Psychology and Neuroscience, Dalhousie University, Halifax, NS, Canada; ^2^Department of Paediatrics, Faculty of Medicine, University of Toronto, Toronto, ON, Canada; ^3^Department of Pediatrics, Faculty of Medicine, Dalhousie University, Halifax, NS, Canada; ^4^Department of Pediatrics, IWK Health Centre, Halifax, NS, Canada; ^5^Departments of Psychology, Family Medicine, and Paediatrics, The University of Western Ontario, London, ON, Canada; ^6^Children's Health Research Institute, London, ON, Canada; ^7^Departments of Pediatrics and Child Health, Faculty of Medicine, University of Manitoba, Winnipeg, MB, Canada; ^8^Faculty of Rehabilitation Medicine, University of Alberta, Edmonton, AB, Canada; ^9^Pediatric Sleep Medicine and Department of Pediatrics, Montreal Children's Hospital, McGill University Health Centre, Montréal, QC, Canada; ^10^Department of Psychiatry and Addictology, Faculty of Medicine, Université de Montréal, Montréal, QC, Canada; ^11^Developmental Pediatrics and Respirology, Faculty of Medicine, University of British Columbia, Vancouver, BC, Canada

**Keywords:** neurodevelopmental disorders (NDDs), insomnia, intervention, behavioral intervention, eHealth, transdiagnostic, barriers and facilitators, children

## Abstract

**Objective:**

Pediatric insomnia is one of the most commonly reported disorders, especially in children with neurodevelopmental disorders. *Better Nights, Better Days for Children with Neurodevelopmental Disorders* (*BNBD-NDD*) is a transdiagnostic, self-guided, eHealth behavioral sleep intervention developed for parents of children with NDDs ages 4–12 years with insomnia. After usability testing, a randomized controlled trial (RCT) was conducted to evaluate the effectiveness of the *BNBD-NDD* program. By interviewing RCT participants after their outcome measures were collected, we sought to determine the barriers and facilitators that affect the reach, effectiveness, adoption, implementation, and maintenance of the *BNBD-NDD* intervention, as well as to assess whether barriers and facilitators differ across levels of engagement with the program and NDD groups.

**Method:**

Twenty parents who had been randomized to the treatment condition of the RCT participated in this study. These parents participated in virtual semi-structured qualitative interviews about their experiences with the *BNBD-NDD* program. Rapid analysis was used, in which one researcher facilitated the interview, and another simultaneously coded the interview using the Reach, Effectiveness, Adoption, Implementation, and Maintenance (RE-AIM) framework.

**Results:**

Overall, more facilitators than barriers were identified for Reach, Effectiveness, Implementation, and Maintenance, whereas for Adoption more barriers emerged. Participants who were engaged reported more facilitators about the *BNBD-NDD* program design and behavior change, while unengaged participants mentioned needing more support to help facilitate their use of the program. Lastly, parents of children with ASD reported more facilitators and more barriers than did parents of children with ADHD.

**Conclusion:**

With this feedback from participants, we can optimize *BNBD-NDD* for large-scale implementation, by modifying the program to better support parents, helping them implement the strategies effectively at home, and increasing the accessibility of this evidence-based treatment.

## 1. Introduction

Pediatric insomnia is one of the most common sleep disorders reported in children (Esposito et al., 2019). In the general population, ~30% of children have symptoms of insomnia, including trouble falling and/or staying asleep, and 5–20% meet the diagnostic criteria for insomnia, including frequent (i.e., three or more times a week) and chronic (i.e., lasting longer than 3 months) difficulties with insomnia symptoms that impair daytime functioning (American Psychiatric Association, 2022). Children with neurodevelopmental disorders (NDDs) are at an even higher risk for insomnia, with up to 90% having insomnia symptoms (Didden and Sigafoos, 2001; Tietze et al., 2012). NDDs are a group of conditions that manifest during the developmental period and result from developmental deficits of the central nervous system that impair one or more areas of functioning (e.g., social, emotional, behavioral).

Insomnia symptoms are associated with increases in daytime sleepiness, fatigue, reduced alertness, and negative effects on cognitive and behavioral functioning and emotional regulation (Sadeh, 2007; Reid et al., 2009; Bub et al., 2011). Insomnia often results in shortened sleep duration, as children cannot adjust their wake times to accommodate longer sleep times due to school. Consequences associated with sleep loss also include increased rates of mental health disorders, deficits in learning and academic performance, and negative effects on physical health, including increased rates of metabolic disorders (Gibson et al., 2006; Palermo et al., 2008). Children with NDDs may be even more vulnerable to the negative effects of poor sleep than their neurotypical peers (Sadeh, 2007). In addition to the above-noted impacts, sleep disturbance may increase NDD symptom severity and impairment, and can interfere with the effectiveness of interventions such as those focused on language development and positive behavioral support (Schreck, 2004; Vriend et al., 2011).

Behavioral factors (e.g., inconsistent bedtime routines, poor sleep practices) are the main contributors to insomnia in typically developing (TD) children and also play a significant role in the etiology of insomnia in children with NDDs (Jan et al., 2008; Vriend et al., 2011). As such, behavioral interventions are the first-line treatment recommended to address insomnia in children, including children with NDDs (Mindell et al., 2006; Blackmer and Feinstein, 2016; Heussler, 2016; Smith et al., 2016). A meta-analysis of 16 randomized controlled trials reviewed behavioral interventions to address insomnia symptoms among children and found that the interventions were overall effective for TD children (Meltzer et al., 2021). While research examining the effectiveness for children with NDDs is limited, the above-noted meta-analysis included three studies that focused on children with NDDs and reported that each study showed statistically significant improvement in sleep efficiency (percentage of time spent asleep in bed) (Meltzer and Mindell, 2004). Despite the strong evidence for the effectiveness of behavioral interventions in TD children and growing evidence for children with NDDs, as well as best-practice guidelines that recommend these interventions as first-line treatment, children's sleep problems are most often treated with medications, especially for children with NDDs and insomnia (Felt and Chervin, 2014; Bock et al., 2016). A primary reason for this is that evidence-based behavioral interventions for children with insomnia are not readily accessible, particularly for children with NDDs.

In recent years, there has been an increase in the amount of research supporting the effectiveness of interventions delivered via the Internet (Weisenmuller and Hilton, 2021). Often called “eHealth interventions”, these can successfully remove barriers, including cost (financial impediments) and accessibility (i.e., living in remote areas, transportation difficulties, long waiting times for access) (Oh et al., 2005; Weisenmuller and Hilton, 2021). Several studies have evaluated Internet-based treatment programs for adult insomnia. A systematic review and meta-analysis, which included 11 randomized control trials (RCTs), reported a significant improvement in sleep parameters among the adult participants who received eHealth interventions (Zachariae et al., 2016). There are no meta-analyses of eHealth for pediatric insomnia, given the small body of research on this topic. However, a recent review indicated that eHealth is an effective way of delivering interventions to treat health challenges in children with pediatric insomnia, mental health problems, and physical disorders, including children with NDDs (Tan-MacNeill et al., 2021).

To bridge the access barriers to treatment for pediatric insomnia, the *Better Nights, Better Days for Typically Developing Children* (*BNBD-TD*) program was developed (Corkum et al., 2018). The *BNBD-TD* program is an eHealth sleep intervention program created for children ages 1–10 years with pediatric insomnia symptoms. A large-scale randomized controlled trial (RCT) of *BNBD-TD* including over 500 parents of TD children with insomnia demonstrated the effectiveness of this intervention in improving children's sleep and daytime functioning, as well as reducing parental fatigue (Corkum et al., 2018). Parents reported high levels of satisfaction with *BNBD-TD*. The *BNBD-TD* program was modified to be appropriate for parents of children with NDD, resulting in *Better Nights, Better Days for Children with Neurodevelopmental Disorders* (*BNBD-NDD*).

An iterative process was undertaken to modify *BNBD-TD* to become *BNBD-NDD*, which was to focus on four highly prevalent NDDs: Attention-Deficit/Hyperactivity Disorder (ADHD), Autism Spectrum Disorder (ASD), Cerebral Palsy (CP), and Fetal Alcohol Spectrum Disorder (FASD). First, a systematic literature review was conducted to determine what consistencies exist in sleep problems across NDD populations and trends in treatment outcomes for children with ADHD, ASD, CP, and FASD (Rigney et al., 2018). Second, a focus group study collected input from parents and healthcare providers on barriers and facilitators to accessing and implementing evidence-based sleep interventions for families of children with NDDs (Tan-MacNeill et al., 2020a). Third, a Delphi study generated input from leading clinicians and researchers in the field of pediatric sleep for children with NDDs to identify the required components of a parent-implemented sleep intervention for children with NDDs (Ali et al., 2018). Fourth, a usability study was conducted with parents of children with NDDs to validate the usability of *BNBD* as a transdiagnostic intervention (Tan-MacNeill et al., 2020b). These four studies supported the development of *BNBD-NDD*—a transdiagnostic, eHealth intervention for children with NDDs who have insomnia.

The resulting *BNBD-NDD* program is a no-cost, self-guided (i.e., there is no contact with coaches or clinicians) five-session program delivered via the Internet that empowers parents to implement strategies independently. The program employs a transdiagnostic approach to treatment for sleep issues common to children with a range of NDDs. Accommodations are also provided to address diagnostic-specific symptoms when appropriate (e.g., how to modify a faded bedtime with response cost procedure for a child with ASD who may be hard to engage with a low interest activity, using visual schedules for bedtime routines for children with ASD and ADHD). The *BNBD-NDD* program delivers interventions that treats pediatric insomnia, while addressing comorbidities often seen in children with NDDs by tailoring the program to each individual child (Corkum et al., 2018).

The effectiveness of the *BNBD-NDD* program for treating insomnia in children 4–12 years old with a diagnosis of ADHD, ASD, FASD, and/or CP and who meet research criteria for insomnia was evaluated through a randomized controlled trial (RCT). Participants were recruited from across Canada and were either assigned to receive the *BNBD-NDD* eHealth program (Intervention group) or to control (Usual Care Group), who do not receive the intervention until completion of the 8-month assessment. The RCT is now complete and a total of 172 families participated in the study. Measures were collected prior to intervention/usual care (baseline), after treatment at 4 months, and at follow-up at 8 months post- randomization. Preliminary analyses of the post-intervention data indicate a high level of parent satisfaction, as well as strong implementation success, including parental reports of effectiveness. However, not all parents engaged with the program, and not all parents found it effective (Vaughan et al., 2022).

The purpose of the present study is to provide insight into how the implementation of the *BNBD-NDD* program could be optimized to increase engagement and effectiveness by understanding what could make the program easier to implement, and what challenges parents faced in using the program. Twenty participants who participated in the aforementioned intervention group were recruited for the present study. More specifically, 13 participants were engaged (i.e., completed three sessions or more) and seven participants were unengaged (i.e., completed zero to two sessions). Data were collected using virtual semi-structured interviews, and content analysis was used to code these interviews using the Reach, Effectiveness, Adoption, Implementation, Maintenance (RE-AIM) framework (Glasgow et al., 1999). This widely used implementation framework evaluates the potential for, or actual, population impact of an intervention studied using either qualitative or mixed methods designs (Holtrop et al., 2018). RE-AIM has been successfully used in research across several mental health domains, as well as community and public health settings, and aligns with our main goals of understanding the facilitators and barriers leading to the reach, effectiveness, implementation, adoption, and maintenance to the *BNBD-NDD* program (Gaglio et al., 2013). Table 1 outlines the dimensions and definitions for the evaluation of the implementation of the *BNBD-NDD* program. With feedback from participants, the researchers plan to modify the *BNBD-NDD* program to better support parents and help them implement the strategies effectively at home with their children.

**Table 1:** RE-AIM implementation framework.

	**Definition**
Reach	An individual-level measure (i.e., patient) of participation. It refers to the percentage and risk characteristics of persons who receive or are affected by a program.
Effectiveness	The impact of an intervention on outcomes, including potential negative effects, quality of life, and economic outcomes.
Adoption	The absolute number, proportion, and representativeness of intervention agents who are willing to initiate a program.
Implementation	Refers to the intervention agents' fidelity to the various elements of an intervention's protocol. This includes consistency of delivery as intended and the time and cost of the intervention.
Maintenance	The individual level is defined as the long-term effects of a program on outcomes 6 or more months after the most recent intervention contact.

This study had three objectives. The first research objective is to determine, irrespective of the child's specific NDD diagnosis, which RE-AIM components represent the most common barriers and facilitators for participants in the *BNBD-NDD* trial, and what parents suggest to improve the intervention for each of the RE-AIM components. The second research objective examines if the most common barriers and facilitators differed based on the parents' degree of engagement with *BNBD-NDD* program (i.e., Unengaged-−0 to 2 sessions, which is considered insufficient engagement with the behavioral intervention strategies to produce clinical change; Engaged—Parents who completed at least 3 sessions of the program). The third research objective examines whether the child's diagnostic group (i.e., ADHD, ASD, FASD, CP) affected the level of program engagement, and the barriers and facilitators that the parent-participant experienced.

## 2. Method

### 2.1. Participants

Eligible participants recruited for this study had been randomized to the intervention condition (i.e., *BNBD-NDD* program), lived in Canada, and had completed their 4-month assessment point (*n* = 86). Participants were not required to have started or completed the program to participate in this study. Participants were ineligible for this study if they were randomized to the usual care (i.e., control) condition or had not completed their 4-month assessment period.

Sampling through computer-generated user statistics collected via the *BNBD-NDD* intervention platform, was used to select parents to invite to take part in this interview based on two conditions related to engagement with the *BNBD-NDD* program: (1) Unengaged: Parents who never accessed the program, accessed the program but did not complete any sessions, or parents who completed the first session or the first two sessions of the program, which was considered insufficient engagement with the behavioral intervention strategies to produce clinical change given that the behavioral strategies to directly address the child's insomnia symptoms were not presented until Session 3; (2) Engaged: Parents who completed at least up to and including Session 3. Parents were also selected based on the primary diagnoses of their children (i.e., ADHD, ASD, CP, FASD), with targeted recruitment designed to fill these four conditions equally. As fewer parents of children with CP and FASD enrolled in the trial, we used purposive sampling and first approached parents of children in these two diagnostic categories to maximize representation. The goal was to recruit a maximum of eight participants for each of the four diagnostic groups (i.e., ADHD, ASD, CP, FASD), with four participants per engagement category (i.e.., engaged and unengaged) within each diagnostic group. While the maximum sample of 32 participants was possible, ongoing analyses were planned with recruitment ending when themes were saturated.

Out of the 85 participants who were contacted to participate for the study, 24 participants consented for the study, and 20 participants completed semi-structured qualitative interviews. Participants were given a $25 Amazon gift card as an Honorarium for participating in the study after their interview was conducted. Given that it was not possible to recruit equal numbers of parents of children for the FASD and CP diagnostic groups to fill the engagement levels, participants were oversampled from alternate diagnostic groups (ADHD and ASD) until saturation of themes was reached. Recruitment was pursued for parents of children that had FASD (*n* = 6) and CP (*n* = 2) until saturation of themes was reached and recruitment was stopped. The final sample comprised 20 participants-−11 participants had children with ADHD, eight participants had children with ASD, and one participant had a child with FASD (see Table 2 for a breakdown of the participant engagement and diagnostic group representation).

**Table 2:** Participants interviewed for the BNBD-NDD B&F study.

**Diagnosis**	**Unengaged participants (*n* = 7)**	**Engaged participants (*n* = 13)**
ADHD (*n* = 11)	5	6
ASD (*n* = 8)	2	6
FASD (*n* = 1)	0	1
CP (*n* = 0)	0	0

Tables 3.1, 3.2 present demographic characteristics for the participants who completed the semi-structured interviews. The average age of parent participants was between 40 and 44 years old. Most participants (*n* = 10, 50%) lived in a city with over 500,000 residents. While the sample included participants from most regions of Canada, the majority (*n* = 10, 50%) were residents of the Central Canadian region (e.g., Ontario, Quebec), as would be expected given the population distribution of Canada. The majority of participants had at least a college/university diploma/certificate (*n* = 18, 90%). Almost all participants were White/Caucasian (*n* = 18, 90%). Half of the participants reported that they had two children living in their home (*n* = 10, 50%). Additionally, the majority of participants reported that they were the biological mother of the child participating in *BNBD-NDD* (*n* = 15, 75%). Most children that were the focus of the *BNBD-NDD* intervention were White/Caucasian (*n* = 15, 75%), and between 10 and 12 years (*n* = 12, 60%).

**Table 3.1:** Demographic details reported by participants.

**Age**	**Total (*N =* 20)**
30–34 years old	2 (10%)
35–39 years old	6 (30%)
40–44 years old	3 (15%)
45–49 years old	7 (35%)
55 years or older	2 (10%)
**Community**	
Rural	3 (15%)
Town	2 (10%)
City under 500,000 people	5 (25%)
City over 500,000 people	10 (50%)
**Region of Canada**	
Central (Ontario, Quebec)	10 (50%)
West (British Columbia, Alberta)	4 (20%)
Atlantic (New Brunswick, PEI, Nova Scotia, Newfoundland, and Labrador)	3 (15%)
Prairie (Saskatchewan, Manitoba)	3 (15%)

**Table 3.2:** Demographic details reported by participants.

**Education**	**Total (*N* = 20)**
Completed high school (or equivalent)	2 (10%)
Diploma or certificate from college, CEGEP, nursing, or university	6 (30%)
Bachelors/undergraduate degree or teacher's college	4 (20%)
Master's degree	6 (30%)
Doctorate	2 (10%)
**Ethnicity**	
White/Caucasian	18 (90%)
Black (e.g., African, Haitian, Jamaican, Somali)	1 (5%)
Other	1 (5%)
**Children living in their home**	
1 child	6 (30%)
2 children	10 (50%)
3 children	3 (15%)
More than 3 children	1 (5%)
**Relationship to child with NDD**	
Biological mother	15 (75%)
Biological father	1 (5%)
Adopted father	1 (5%)
Adopted mother	2 (10%)
Grandmother	1 (5%)
**Child's ethnicity**	**Total (** ***N** **=** **20)***
White/Caucasian	15 (75%)
Aboriginal	2 (10%)
Black (e.g., African, Haitian, Jamaican, Somali)	1 (5%)
Bi-racial/mixed-race	2 (10%)
**Child's age (at time of study)**	
4–5 years old	1 (5%)
6–9 years old	7 (35%)
10–12 years old	12 (60%)

### 2.2. Procedures

The Research Electronic Data Capture (REDCap; Harris et al., 2019), a secure online platform, was used to deliver all surveys and communications. We sent an email to parents inviting them to participate and directed them to the consent form. If participants did not respond to the initial invitation, they received three email reminders and up to two telephone reminders. Parents who consented to participate in this study were directed to a survey to schedule their interview appointment through REDCap. Participants received two reminder emails 24 and 2 h before their interview.

At the time of the interview, the relevant consent information was summarized by the interviewer, and participants had an opportunity to ask questions. Virtual semi-structured interviews, lasting 20–30 min each, were conducted without video and were audio-recorded and transcribed using the web-based Microsoft Teams software. Two research team members were involved in each interview. One researcher conducted the interview (Researcher A; A.I.), and one researcher (Researcher B; M.O.) simultaneously rapid coded the interview (see Appendix A) (Taylor et al., 2018; Gale et al., 2019; Vest et al., 2020). QSR International's NVivo 12 qualitative data analysis software was used to organize the data in the 20 interviews for analysis (QSR International, 1999). For more details about analysis, see Section 2.4 below.

### 2.3. Measures

#### 2.3.1. Demographic questionnaire

Information from the demographic questionnaire completed during the baseline measures for the RCT was accessed to describe the demographic and socio-economic information about the participant, their spouse/partner, and the child who was the focus of the intervention the sample. The questionnaire also collected information on the region where the participants lived in Canada (e.g., Western Canada—British Columbia and Alberta; Prairies—Saskatchewan and Manitoba; Central Canada—Ontario and Quebec; Eastern Canada—New Brunswick, Nova Scotia, Prince Edward Island, and Newfoundland and Labrador; Territories—Yukon, Northern Territories, and Nunavut). This 32-item questionnaire was author-made, based on the Canadian Consensus and the National Longitudinal Survey of Children and Youth (Canadian National Longitudinal Survey of Children Youth, 2011).

#### 2.3.2. Interview guide

The interview guide (see Appendix A) is a researcher-created measure modeled after an interview schedule used to explore the barriers and facilitators of an eLearning platform to deliver professional development to teachers working with children with NDDs (Ali et al., 2020). The guide included 23 open-ended questions designed to elicit information about parents' experiences of barriers and facilitators related to the *BNBD-NDD* program (e.g., did they find the program effective, what contributed/interfered with the effectiveness of the program, were participants able to implement the program's strategies). Furthermore, we asked participant's questions about sustainability of the program, including the possibility of purchasing the program if commercialized. Since these questions are outside of the scope of implementation, the results will be reported elsewhere. There are also three questions at the end of the interview guide that asked parents: (a) if they would be interested in potentially using updated versions of the program in the future, (b) how the researchers could engage more parents with the program, and (c) if they have any remaining questions or comments.

### 2.4. Data analysis

Rapid coding involved categorizing all unique ideas or concepts within the interview responses, with each response being placed under at least one element of the RE-AIM framework (Glasgow et al., 1999). To summarize interview responses, a templated summary table, based on the methods used in previous qualitative rapid analysis studies, was used (Taylor et al., 2018; Gale et al., 2019). Please see Appendix A for a copy of the table. Each templated summary table completed during rapid analysis summarized the participant interviews, the relevant RE-AIM themes that were discussed, and barrier and facilitator numerical ratings. Once the interviews were summarized, the summaries were consolidated by participant type. Immediately after the interview had been conducted, Researcher A (A.I.) and Researcher B (M.O.) met and reviewed the interview audio recording and Researcher B's notes to ensure consistency. During this time, each researcher independently reviewed the interview summary and reviewed the barriers and facilitators, using separate templated summary tables. After this review, Researchers A and B resolved any inconsistencies by discussion and finalized the interview summary sheet. The finalized and agreed upon interview summaries, as well as the barriers and facilitators, were collated into the summary table for each RE-AIM element and indicated which elements received which barrier and facilitator ratings and provided any required contextual notes for interpretation. Finally, a third researcher (Researcher C; H.S.) listened to the recording while reviewing the templated summary table from the interview to identify any discrepancies. The percentage of coding agreement between the coders was 90%. Discrepancies were resolved by discussing them with a senior member of the research team (P.C.).

Once the rapid coding and analysis was complete, qualitative data were further analyzed using content analysis (Hsieh and Shannon, 2005; Schreier, 2012). The content analysis involved five stages: (1) familiarization with the data, (2) generation of initial codes, (3) search for themes, (4) review identified themes, and (5) definition and naming of the themes (Hsieh and Shannon, 2005). The participant summaries were compared by two coders (A.I. and M.O.) to develop themes that describe potential barriers to and facilitators of the program's implementation. These themes were discussed with a senior member of the research team (P.C.) to build rigor and trustworthiness. Each sub-theme had to be identified by three or more participants to be included in the larger themes for each element of the RE-AIM framework, based on an a priori for threshold inclusion of 15% (3/20), consistent with the approach of Ali et al. (2020).

## 3. Results

### 3.1. Research Objective 1: Irrespective of the child's specific NDD diagnosis, which RE-AIM components represent the most common barriers and facilitators for participants in the BNBD-NDD trial, and what parents suggest to improve the intervention for each of the RE-AIM components

Described below, and in Supplementary Tables 1.1–1.10, are the themes and sub-themes related to facilitators and barriers for each of the five RE-AIM components, along with the participants' recommendations to enhance each. Overall, 19 themes were identified, with 47 subthemes that reached criteria (15%; three individual participants mentioned). To be inclusive of all themes, sub-themes that did not meet criteria were included in the Supplementary material. Each theme that reached inclusion criteria is described in turn, below.

#### 3.1.1. Reach

For the Reach component of the RE-AIM framework, four themes were identified. Motivation to Participate, Program Discovery, and Credibility were facilitating themes, and Time Commitment was a barrier theme (see Supplementary Table 1.1). Within these four themes, 15 sub-themes met the inclusion criteria (14 facilitators, one barrier). All (*n* = 20) participants stated that their child's sleep problems motivated them to participate in the program. Most participants (*n* = 12) stated that they discovered the *BNBD-NDD* program through healthcare providers, healthcare centers, schools, Facebook, support groups, and NDD organizations. A few participants (*n* = 3) mentioned that the program seemed credible, which appealed to them. Time commitment was mentioned by some participants as a factor that made them hesitant to participate in the program (*n* = 7). The most frequent suggestions for improving the Reach of *BNBD-NDD* were advertising in parent support groups, healthcare centers, schools, NDD organizations, and reaching out to healthcare providers for program referrals (see Supplementary Table 1.2).

#### 3.1.2. Effectiveness

For the Effectiveness component, four themes were identified that met threshold. Program Design Facilitators and Behavior Change Success were facilitating themes, and Contextual Factors and Suggested Improvements to Program Design were barrier themes (see Supplementary Table 1.3). Within these themes, six sub-themes met the inclusion criteria (four facilitators, two barriers). The majority (*n* = 12, 60%) of participants stated that the program was effective and noted success in improving their child's sleep. These results need to be interpreted with caution, as some participants who answered questions regarding effectiveness had no engagement with the program. Effectiveness was thought to be influenced by the way the program was structured, the ability to tailor the program to the needs of the child, and by creating a bedtime routine. Contextual factors that were barriers to program effectiveness were mentioned by participants (*n* = 11, 58%), and included circumstances beyond parent's control, such as unanticipated life challenges (e.g., family members getting sick, competing demands of multiple children) and challenges related to the COVID-19 pandemic (e.g., switching from in-person to online learning). The other barrier theme was related to program design; some (*n* = 3, 15%) participants mentioned that the program structure reduced effectiveness (e.g., sessions too long, not being able to pick which sessions to complete). The suggestions most mentioned to increase program Effectiveness were being able to skip through sessions, having or creating a routine to review the program and implement strategies, modifying the program length, and increasing individual tailoring (see Supplementary Table 1.4).

#### 3.1.3. Adoption

For the Adoption dimension, two themes were identified. Program Refinement, and Program Access were barrier themes (see Supplementary Table 1.5). Within these themes, 10 sub-themes met the inclusion criteria. The structure of the program (e.g., having to complete modules in sequential order) (*n* = 8, 40%), time commitment, (*n* = 5, 25%), lack of coaching (*n* = 5, 25%), challenges with tailoring the program to the child's needs (e.g., not tailored enough for their child) (*n* = 4, 20%), and the lack of scientific evidence of the effectiveness of the program (*n* = 3, 15%) were reported as barriers that could be addressed during program refinement. Lastly, parents perceived the lack of users' testimonials (*n* = 7, 35%), limited knowledge upfront of program structure/content (*n* = 6, 30%), lack of referrals by healthcare providers (*n* = 5, 25%) and healthcare centers (*n* = 3, 15%), and word-of-mouth (*n* = 3, 15%) as barriers for the program's adoption. The most frequent Adoption suggestions were having evidence of effectiveness/testimonials from parents who had completed the program, adding coaching/parent support to the program, healthcare providers recommending the program, and having knowledge of program structure/content before starting the program (see Supplementary Table 1.6).

#### 3.1.4. Implementation

For the Implementation component, five themes were identified. Level and Timing of Implementation, Program Design Facilitators, and Factors Supporting Implementation were facilitating themes, and Contextual Factors and Program Design Barriers were barrier themes (see Supplementary Table 1.7). Within these themes, 13 sub-themes met the inclusion criteria (nine facilitators, four barriers). For level and timing of Implementation, most participants (*n* = 11, 55%) stated they were able to implement the strategies recommended by the program in their children's lives, and some participants (*n* = 9, 45%) stated they implemented these strategies daily. Participants noted sleep problems in their children as a motivator for implementing the strategies (*n* = 5, 25%), and most participants (*n* = 10, 50%) stated they implemented at least some of the strategies. For program design facilitators, the structure of the program (i.e., session content, session timing) was the most frequently mentioned sub-theme (*n* = 4, 20%). Factors supporting implementation included spousal support and their child's behavior (e.g., being cooperative). For barriers to implementation, contextual factors such as their child's behavior (e.g., presence of disruptive behavior), circumstances beyond their control, and parental exhaustion were mentioned. The structure of the program (e.g., length of sessions) was most frequently mentioned as a sub-theme for program design barriers (*n* = 6, 30%). The most mentioned suggestions for Implementation were adding coaching/support and having additional reminders to help parents with program adherence (see Supplementary Table 1.8).

#### 3.1.5. Maintenance

For the Maintenance component, two themes were identified. Using Tools and Resources was a facilitating theme, and Not Using Tools and Resources was a barrier theme (see Supplementary Table 1.9). Within these themes, three sub-themes met the inclusion criteria (two facilitators, one barrier). Of the 20 participants, 11 (55%) participants reported using the tools and resources from the program after completing the program. But, one in four participants (*n* = 5, 25%) did not refer to the program's resources and tools after program completion; this was a barrier to the program effectiveness for these participants. Most Maintenance suggestions were for reminders after completion of the program to help parents with continued use of strategies, access to the *BNBD-NDD* program for a longer time (e.g., 6 months to a year), modifying the length of program access on a case-by-case basis, coaching/support for participants, and tangible tools to support program content (e.g., printouts, booklets) (see Supplementary Table 1.10).

### 3.2. Research Objective 2: Examine whether the most common barriers and facilitators differed based on the parents' degree of engagement with BNBD-NDD program

Described below, and in the Supplementary Tables 2.1–2.5, are the themes related to facilitators and barriers for engagement based on two categories: Engaged (i.e., participants who completed three or more sessions of the program) and Unengaged (i.e., participants who completed zero to two sessions of the program) (see Table 2 for participant breakdown). For reporting, we are using a threshold of 15%, where we only report differences that are 15% or higher to identify barrier and facilitator themes as related to engagement.

Differences were found between the two levels of engagement for all RE-AIM dimensions on most of the sub-themes. For Reach, only engaged participants (38%) reported credibility as a facilitator, and engaged participants reported time commitment as a barrier (46%) more than unengaged participants (29%). For Effectiveness, engaged participants reported more facilitating sub-themes compared to unengaged participants, such as program design facilitators (85 and 43%) and behavior change success (92 and 43%). Examples include the program's step-by-step approach and being able to review videos and sessions that they already completed. Participants who were engaged noted more contextual factors (69%) and improvements to program design (54%) than participants who were unengaged (29 and 14%, respectively). Examples included child's medication, family stress (e.g., family issues, siblings), COVID-19 (i.e., pandemic lockdowns), family schedules (e.g., school, sickness, appointments) and having to review the whole program (i.e., not being able to pick and choose what sessions are most relevant).

For Adoption, engaged participants mentioned more program access barriers (92%) than unengaged participants (57%). Examples included spreading information about the program through word-of-mouth and having healthcare provider recommendations. For Implementation, participants who were engaged mentioned more program design facilitators (38%) and factors supporting implementation (46%) than unengaged participants (14 and 14%, respectively). Examples include creating a bedtime routine centered around the program's strategies, tracking their child's sleep with daily sleep diaries, doing the program during the summer, and having another parent helping with implementing the strategies. In contrast, unengaged participants mentioned more program design barriers (86%), such as program length and having the program more tailored to individual participants than engaged participants (46%). For Maintenance, engaged participants mentioned more facilitators to using program tools and resources (92%) compared to unengaged participants (29%). Examples include using strategies and resources from the program after completing *BNBD-NDD*.

There were no differences between engaged and unengaged participants on a number of the subthemes within four RE-AIM dimensions, including Reach (motivation to participate), Adoption (program refinement), Implementation (level and timing of implementation and contextual factors), and Maintenance (not using tools and resources).

### 3.3. Research Objective 3: Examine whether the child's diagnostic group (i.e., ADHD, ASD, FASD, CP) affected the level of program engagement and barriers and facilitators that the parent-participant experienced

Described below, and in the Supplementary Tables 3.1–3.5, are the facilitators, barriers, and engagement level (e.g., Unengaged-−0 to 2 sessions; Engaged-−3 to 5 sessions), as well as similarities and differences for two groups (ADHD and ASD). FASD and CP have been excluded from this analysis, as only one interview participant had a child with FASD and no parents of children with CP participated. For reporting, we are using a threshold of 15%, where we only report differences that are 15% or higher to determine differences between diagnostic groups for barrier and facilitator themes. The level of engagement differed between the ADHD (*n* = 11; 46% Unengaged, 54% Engaged) and ASD (*n* = 8; 25% Unengaged, 75% Engaged) diagnostic groups (see Table 2).

Differences were found between the two diagnostic groups for most of the sub-themes on four of the five RE-AIM dimensions. For Reach, parents of children with ASD reported more facilitators related to the credibility of the program (38%) compared to parents of children with ADHD (18%), noting that the credibility of the program made them more motivated to participate. For Effectiveness, parents of children with ASD, compared the parents of children with ADHD, reported more facilitators related to program design (88 and 55%, respectively), indicating that the program being presented in simple steps and using sleep diaries contributed to the program's effectiveness. They also reported more barriers, including contextual factors (75 and 45%, respectively) and improvements to program design (50 and 27%, respectively). These included being too busy/exhaustion, external life factors, shortened program sessions, and adding coaching to the program.

For Adoption, participants who had children with ADHD, compared to parents of children with ASD, reported more barriers related to program refinement (82 and 50%, respectively). Examples of program refinement included not tailored enough to their family's needs and too much of a time commitment. For Implementation, they reported more program design barriers than participants who had children with ASD (73 and 50%, respectively). Examples of program design barriers included length of the program and the sessions not being released all at once (i.e., having to wait a week to access the next program session). Parents of children with ADHD also noted more facilitators to level and timing of implementation (90%) compared to parents of children with ASD (63%). These included using the program's strategies immediately and using the program's strategies every night.

There were no differences between the ADHD and ASD groups for the following sub-themes, within the RE-AIM dimensions of Reach (motivation to participate and time commitment), Effectiveness (behavior change success), Adoption (program access), Implementation (program design facilitators, factors supporting implementation), and Maintenance (using program tools and resources after they completed the program).

## 4. Discussion

The purpose of this study was to evaluate how parents of children with NDDs perceived the barriers and facilitators of the *BNBD-NDD* insomnia treatment program, using the RE-AIM framework for analysis, and to report participants' suggested modifications to improve the program. Overall, all parents (collapsed across engaged, non-engaged, and NDD groups) reported more facilitators than barriers regarding the *BNBD-NDD* program, regardless of their engagement level in the program or their children's NDD diagnosis. The most frequently mentioned suggestions for improving the program were adding coaching support to help parents act on the strategies provided by the program, adding testimonials and evidence of program's effectiveness to its website, sending reminders to participants after program completion to review the program materials, and continuing to increase the program's reach through advertising to parent support groups, healthcare centers, and schools. Results show that participants that were more engaged in the program reported the most benefits, and reported not only more facilitators, but also more barriers to the program's implementation compared to those who were unengaged. However, the unengaged group did report more barriers related to the design of the intervention compared to the engaged group. Overall, parents of children with ASD generally reported more facilitators and more barriers than parents of children with ADHD.

The first research objective evaluated the specific elements of the RE-AIM framework and whether they were barriers or facilitating factors in parents' engagement in the *BNBD-NDD* intervention. The Reach, Effectiveness, Implementation, and Maintenance elements of the RE-AIM framework were perceived as having more facilitators than barriers, while Adoption was perceived more as a barrier than a facilitating factor. This is positive, as it means that generally the program was perceived to have strong reach to parents who need such a program and the strategies suggested by the program were readily implementable by parents. Most parents also reported it to be effective in improving their children's sleeping habits and behaviors and were able to maintain these positive changes over time (e.g., less difficulty falling asleep, children staying asleep/not waking up as much more frequently). Parents' perceptions of the program's Reach as a facilitator is not surprising, given the scarcity of behavioral sleep interventions for children diagnosed with NDDs and the fact that most families in Canada do not have access to treatment for pediatric insomnia and that many healthcare providers are not trained in this area. In addition, parents perceived the program as relatively straightforward to implement. Potentially the fact that it was developed using a user-centered design and that parent input was incorporated at each stage of its development (Ali et al., 2018; Rigney et al., 2018; Tan-MacNeill et al., 2020a,b) may have resulted in a program that was perceived as effective. Furthermore, given that the program was developed based on scientific evidence that address pediatric insomnia in children with NDDs (Mindell et al., 2006; Corkum et al., 2019), it is not surprising that parents perceived the program to be effective and that positive changes in the child's sleep habits were maintained over time. The main challenge with the program appears to be with its adoption/uptake. It is possible that the already high levels of stress in families with children with NDDs, combined with challenges associated with the COVID-19 pandemic at the time of the study (Adams et al., 2021) may have contributed to challenges in families' adoption of this program.

Parents were also asked for suggestions to improve the *BNBD-NDD* program. Suggestions to improve reach included sharing information about *BNBD-NDD* with parent support groups and healthcare providers, advertising in healthcare centers, schools, and NDD organizations, as well as allowing users of the program to skip through program sessions that the parent may feel less applicable or helpful to their child's situation. Including evidence of effectiveness and participant testimonials and encouraging healthcare provider program recommendations were suggestions to help facilitate program uptake. Adding coaching/support for parents in the program and including program reminders for parents were also recommended. Participants indicated that reminders to refer to the program after completion, extending program access past 6 months, assigning program access on a case-by-case basis, and adding more tangible tools for its implementation may be possible avenues to facilitate maintenance of the program. It is important to note that some of the changes suggested by participants are already a part of the program (e.g., reminders, tangible tools). It raises the question of how to make these features more salient to future participants. Indeed, these design components have been found to help implementation in other programs that moved research into practice (Shaw et al., 2019).

The second research objective evaluated whether the most common barriers and facilitators identified by parents differed based on their degree of engagement with the *BNBD-NDD* program. Overall, unengaged parents reported fewer facilitators and fewer barriers compared to engaged parents. An exception was Implementation, for which unengaged participants reported more barriers related to program design (e.g., being delivered online) than engaged participants. This might be explained by a lesser preference toward online learning by these parents. Indeed, recent research found that more than one-third of parents of children diagnosed with ADHD described remote learning as very challenging, and struggled to support their children at home, especially during the COVID-19 pandemic (Becker et al., 2020; He et al., 2021). Furthermore, parents who did not engage in the program did not mention more barriers to participating in the program than participants who were engaged. It is possible that their lack of participation may be due to factors not measured in this study (e.g., personality, locus of control, global pandemic challenges) (Vollrath and Torgersen, 2008; He et al., 2021). For example, inclination toward certain health-promoting behaviors is closely related to individual personality types, such as higher levels of openness to experience (Vollrath and Torgersen, 2008). Other factors that have not been assessed in this study may have also played a role. We know from previous studies that parental involvement, monitoring, goals, values, and aspirations in relation to their children's adoption of new habits are affected by a range of factors and considerations, including socio-economic status, parenting style, the child's temperament, and an overall warm and nurturing connection between parent and child (Whitebread and Basilio, 2012; Bowling et al., 2019). Future studies should attempt to evaluate factors that may contribute to either engagement or avoidance behaviors leading to new sleep habit formation.

The third research objective evaluated whether the most common barriers and facilitators differed based on the NDD group of the participant's child. Because only one FASD participant and no CP participants participated in the interviews, these diagnostic groups were not included in this analysis, so only ADHD and ASD were included. Of the 19 themes, ADHD and ASD were similar for 10 themes. For the remaining nine themes, parents of children who were diagnosed with ASD generally reported more facilitators and more barriers compared to parents of children diagnosed with ADHD. Specifically, credibility (Reach), program design facilitators, contextual factors, suggested improvements to program design (Effectiveness), program refinement (Adoption), and contextual factors (Implementation) were more frequently reported by parents of children who were diagnosed with ASD. Higher criteria for implementation of the program by parents of children diagnosed with ASD may reflect a higher threshold of scrutiny and credibility of the resources recommended to this group to counteract the challenges parents may face in implementing recommendations for their children. Furthermore, the differences in diagnostic groups could have been influenced by the differing levels of engagement between ASD (25% unengaged, 75% engaged) and ADHD (45% unengaged, 55% engaged).

This study is not without limitations. We were not able to recruit our desired sample size of CP and FASD participants, despite purposive sampling. This stemmed from both small numbers of CP and FASD participants in the RCT, and lack of parental responding to recruitment emails and phone calls. Other limitations are related to the sample and methods. Our sample were predominantly Caucasian and educated (i.e., most of the sample attended College or University), which limits our ability to generalize these findings. Moreover, the questions asked of participants in the semi-structured interview could be seen as leading in terms of identifying barriers and not facilitators (e.g., what interfered with effectiveness, what hindered you from implementing the strategies). In addition, we did not ask participants about the severity of their child's sleep problems during the interview. This may have been correlated with participant's engagement level. Lastly, we chose thresholds for each research question to discuss notable differences between barriers, facilitators, engagement level, and diagnostic groupings (15%). Given the lack of empirical evidence to provide direction on this, having a reasonable a priori threshold, which has been used previously in this type of research (Ali et al., 2021), seemed like the best course of action to discuss differences and similarities between themes, barriers, facilitators, diagnostic groups, and engagement level.

The current study has strengths in both design and real-world implications. To our knowledge, this is the first study to examine the barriers and facilitators in an eHealth intervention program for children with NDDs and pediatric insomnia. Previously, healthcare providers and parents of children with NDDs and pediatric insomnia were interviewed and it was found that face-to-face treatment was not an accessible or feasible option to some parents (Tan-MacNeill et al., 2020a). The online delivery of *BNBD-NDD* has been designed to address these barriers (Corkum et al., 2018). Additionally, parent-implemented interventions may reduce healthcare provider wait times and provide faster solutions to parents of children with NDDs with sleep problems (Althoff et al., 2019). Making sure that the *BNBD-NDD* program is reaching as wide an audience as possible (i.e., parents with children with NDDs, healthcare providers), adding support to the program (i.e., coaching, parent support groups), and more flexibility for parents navigating the program (i.e., extending access time) are priorities that will be addressed in the *BNBD-NDD* program. By increasing the accessibility of evidence-based treatment, the *BNBD-NDD* program may empower parents to improve their children's sleep, thereby increasing the efficiency of NDD care, promoting healthy sleep habits, and improving clinical outcomes and quality of life for children with NDDs and their families.

In summary, parents perceived that the *BNBD-NDD* program was implementable and effective, and they identified many facilitating factors in terms of the program's adoption. This was true for parents of children with ASD and with ADHD, thus supporting the transdiagnostic approach of the program (Tan-MacNeill et al., 2020a). However, not all participants were engaged, and several barriers were reported, particularly related to the adoption (i.e., utilization) of the program. Perhaps some of the parents' lack of engagement and barriers to the program can be understood through the Integrative Model of Change model developed by Prochaska and DiClemente (1983). While it was likely that all participants were at least in the Contemplation stage, or they would not have joined the study, it is also possible that not all participants were in the Preparation and Action stages. To increase uptake of the *BNBD-NDD* program, participants need to be able to move ahead from the Preparation stage to the Action stage. Inclusion of motivational interviewing techniques to help move parents into the Action stage and adding coaching or support into the program may be helpful to increase program adoption (Hall et al., 2012). Suggestions pertaining to the RE-AIM elements will be used to modify *BNBD-NDD* for the upcoming Implementation study. Parents who might otherwise show lower engagement may benefit from more support to facilitate program adherence, enabling them and their children to benefit more from the intervention.

## Data availability statement

The datasets presented in this article are not readily available because it is qualitative data that was collected via interviews. Therefore, we cannot release any data as it may be identifiable. Requests to access the datasets should be directed to PC, penny.corkum@dal.ca.

## Ethics statement

The studies involving human participants were reviewed and approved by IWK Ethics Review Board. The participants provided their written informed consent to participate in this study.

## Author contributions

Conceptualization: MO and PC. Data management, analytic plan, and data analyses: AI, MO, and PC. Interpretation: AI and PC. Writing—original draft preparation: AI. Writing—review and editing: AI, MO, PC, SW, CB, AH-D, OI, EC, RG, GR, SS, and IS. Supervision of all aspects of the research and manuscript process: PC. All authors have read and agreed to the published version of the manuscript.
