# Supplementary material for: Optimizing the Better Nights, Better Days for Children with Neurodevelopmental Disorders program for large scale implementation
Source: Front Sleep. 2023 May 4;2:1158983. doi: 10.3389/frsle.2023.1158983 (PMC12713869; doi:10.3389/frsle.2023.1158983)
Supplement: Supplementary file 1 [file Data_Sheet_1.PDF]

Supplementary Material

eTable 1.1

Reach Themes identified as Barriers or Facilitating Factors (number of participants who identified them out of 20, frequency of facilitators or barriers identified from total identifications)

Facilitators (*N* = 20, 100%)	Barriers (*N* = 20, 100%)
**1. Motivation to Participate (20, 44.5%)**	**1. Time Commitment (8, 100%)**
1.1 Sleep Problems (20)	1.1 Time Commitment (7)
	1.2 Structure of program (1)
**2. Program Discovery (20, 44.5%)**	
2.1 Healthcare Provider (9)	
2.2 Healthcare Centres (6) and Schools (6)	
2.3 Facebook (5)	
2.4 Support group (4) and Other Methods of Discovery (4) and NDD Organizations (4)	
2.5 Visually appealing (3) and Printed Ads (3) and Website (3) and Social Media (3)	
2.6 Word-of-mouth (2)	
**3. Credibility (5, 11%)**	
3.1 Credible (3)	
3.2 Accessibility (1)	
3.3 Tailoring (1)	

Note: REACH sub-themes (number of participants out of the total sample of 20 participants). Percentages are the percent for facilitators and barriers separately (i.e., columns add up to 100%).

eTable 1.2

Reach Suggestions (percentage of suggestions based on the total number of suggestions)

	**Suggestions**
Advertise in Parent Support Groups	5 (23%)
Advertise at Healthcare Centres	5 (23%)
Advertise in Schools	4 (18%)
Advertise in NDD Organizations	4 (18%)
Reach out to Healthcare Providers	4 (18%)

eTable 1.3

Effectiveness Themes identified as Barriers or Facilitating Factors (number of participants who identified them out of 20, frequency of facilitators or barriers identified from total identifications)

Facilitators (*N* = 20, 100%)	Barriers (*N* = 20, 100%)
**1. Program Design Facilitators (14, 52%)**<br>1.1 Structure of Program (9)<br>1.2 Tailoring (4)<br>1.3 Routine – Having One or Creating One (3)<br>1.4 Psychoeducation (2) and Tangible Tools (2) and Videos (2)<br>1.5 Accessibility (1) and Coaching (1) and Credible (1) and Knowledge or Program Structure or Content (1) and Reminders (1) and Roadblocks (1) and Sleep diary (1) and Testimonials (1)	**1. Contextual Factors (11, 58%)**<br>3.1 Circumstances Beyond Control (9)<br>3.2 Child's behaviour (2) and COVID-19 (2)<br>3.3 Time Commitment (1) and Sleep Medication (1) and Multiple Subjects and/or Diagnoses (1)<br><br>**2. Suggested Improvements to Program Design (8, 42%)**<br>2.1 Structure of Program (3)<br>2.2 Parent testimonial videos (2) and Videos (2)<br>2.3 Coaching (1) and Tailoring (1)
**2. Behaviour Change Success (13, 48%)**<br>2.1 Program was Effective (12)	

Note: EFFECTIVENESS sub-themes (number of participants out of the total sample of 20 participants). Percentages are the percent for facilitators and barriers separately (i.e., columns add up to 100%).

eTable 1.4

Effectiveness Suggestions (percentage of suggestions based on the total number of suggestions)

	**Suggestions**
Being able to skip through sessions	4 (29%)
Routine	4 (29%)
Program Length	3 (21%)
Program Tailoring	3 (21%)

eTable 1.5

Adoption Themes identified as Barriers or Facilitating Factors (number of participants who identified them out of 20, frequency of facilitators or barriers identified from total identifications)

Facilitators (*N* = 20, 100%)	Barriers (*N* = 20, 100%)
	**1. Program Refinement (14, 32%)**<br>1.1 Structure of Program (8)<br>1.2 Time Commitment (5) and Lack of Coaching (5)<br>1.3 Challenges to Tailoring (4)<br>1.4 Lack of Evidence of Effectiveness (3)<br>1.5 Content updates (2)<br>1.6 Accessibility (1) and Credible (1) and Tangible tools (1) and Technical Difficulties (1) and Videos (1)<br><br>**2. Program Access (16, 36%)**<br>3.1 Lack of User Testimonials (7)<br>3.2 Limited Knowledge of program structure or content (6)<br>3.3 Lack of Referrals to Healthcare provider (5)<br>3.4 Lack of Referrals to Healthcare centres (3) and Word-of-mouth (3)<br>3.5) Advertisements (2) and NDD Organizations (2)<br>3.6 Schools (1) and Support Group (1)

Note: ADOPTION sub-themes (number of participants out of the total sample of 20 participants). Percentages are the percent for facilitators and barriers separately (i.e., columns add up to 100%).

eTable 1.6

Adoption Suggestions (percentage of suggestions based on the total number of suggestions)

	Suggestions
Evidence of Effectiveness/Testimonials	10 (40%)
Coaching/Added Support	6 (24%)
Healthcare Provider Recommendations	5 (20%)

Knowledge of Program Structure/Content	4 (16%)

eTable 1.7

Implementation Themes identified as Barriers or Facilitating Factors (number of participants who identified them out of 20, frequency of facilitators or barriers identified from total identifications)

Facilitators (*N* = 20, 100%)	Barriers (*N* = 20, 100%)
**1. Level and Timing of Implementation (16, 55%)**<br>1.1 Able to implement (11)<br>1.2 Daily (9)<br>1.3 Sleep problems (5)<br>1.4 Consistent implementation (4)<br>1.5 Immediately implemented (3) and Some Implementation (3)<br>1.6 Implemented in 1-2 weeks (1) and Implemented within a month (1)	**1. Contextual Factors (13. 52%)**<br>1.1 Child's Behaviour (5)<br>1.2 Circumstances Beyond Control (4)<br>1.3 Exhaustion (3)<br>1.4 Multiple subjects and/or diagnoses (2) and Time Commitment (2)<br>1.5 COVID-19 (1) and Spouse (1)
**2. Program Design Facilitators (6, 21%)**<br>2.1 Structure of Program (4)<br>2.2 Accessibility (2) and Sleep Diary (2) and Videos (2)<br>2.3 Psychoeducation (1) and Roadblock (1) and Routine (1) and Tangible tools (1)	**2. Program Design Barriers (12, 48%)**<br>2.1 Structure of Program (6)<br>2.2 Tailoring (2) and Tangible tools (2) and Technical difficulties (2) and Videos (2)<br>2.3 Accessibility and Parent testimonial videos (1) and Roadblocks (1) and Routine (1)
**3. Factors Supporting Implementation (7, 24%)**<br>3.1 Spouse (3) and Child's Behaviour (3)<br>3.2 Work schedule (2)<br>3.3 Circumstances beyond control (1) and COVID-19 (1) and Family (1) and Time commitment (1)	

Note: IMPLEMENTATION sub-themes (number of participants out of the total sample of 20 participants). Percentages are the percent for facilitators and barriers separately (i.e., columns add up to 100%).

eTable 1.8

Implementation Suggestions (percentage of suggestions based on the total number of suggestions)

	Suggestions
Coaching	7 (70%)
Reminders	3 (30%)

eTable 1.9

Maintenance Themes identified as Barriers or Facilitating Factors (number of participants who identified them out of 20, frequency of facilitators or barriers identified from total identifications)

Facilitators (*N* = 20, 100%)	Barriers (*N* = 20, 100%)
**1. Using Tools and Resources (14, 100%)**<br>1.1 Uses most or all strategies (11)<br>1.2 Did refer back to program (7)<br>1.3 Reminders (1) and Tailoring (1) and Tangible Tools (1) and Uses Some Strategies (1)	**1. Did Not Use Tools and Resources (6, 100%)**<br>1.1 Did not refer back to program (5)<br>1.2 Does not use strategies (1)

Note: MAINTENANCE sub-themes (number of participants out of the total sample of 20 participants). Percentages are the percent for facilitators and barriers separately (i.e., columns add up to 100%).

eTable 1.10

Maintenance Suggestions (percentage of suggestions based on the total number of suggestions)

	Suggestions
Reminders	11 (37%)
Program Access – 6 months to 1 year	7 (23%)
Program Access – Case-by-case basis	5 (17%)
Coaching	4 (13%)
Tangible Tools	3 (10%)

eTable 2.1

Reach Themes by participant engagement level (number of participants who identified them, percentage of engagement based on the total number of participants who identified each theme)

	Engaged (*n* = 13)	Non-Engaged (*n* = 7)
Motivation to Participate (F)	13 (100%)	7 (100%)
**Time Commitment (B)**	**6 (46%)**	**2 (29%)**
**Credibility (F)**	**5 (38%)**	**0**

Note: F – Facilitator, B – Barrier; Bolded numbers and percentages are sub-themes that met the 15% difference threshold.

eTable 2.2

Effectiveness Themes by participant engagement level (number of participants who identified them, percentage of engagement based on the total number of participants who identified each theme)

	Engaged (*n* = 13)	Non-Engaged (*n* = 7)
**Program Design Facilitators (F)**	**11 (85%)**	**3 (43%)**
**Behaviour Change Success (F)**	**12 (92%)**	**3 (43%)**
**Contextual Factors (B)**	**9 (69%)**	**2 (29%)**
**Suggested Improvements to Program Design (B)**	**7 (54%)**	**1 (14%)**

Note: F – Facilitator, B – Barrier; Bolded numbers and percentages are sub-themes that met the 15% difference threshold.

eTable 2.3

Adoption Themes by participant engagement level (number of participants who identified them, percentage of engagement based on the total number of participants who identified each theme)

	Engaged (*n* = 13)	Non-Engaged (*n* = 7)
Program Refinement (B)	9 (69%)	5 (57%)
**Program Access (B)**	**12 (92%)**	**4 (57%)**

Note: F – Facilitator, B – Barrier; Bolded numbers and percentages are sub-themes that met the 15% difference threshold.

eTable 2.4

Implementation Themes by participant engagement level (number of participants who identified them, percentage of engagement based on the total number of participants who identified each theme)

	Engaged (*n* = 13)	Non-Engaged (*n* = 7)
Level and Timing of Implementation (F)	10 (77%)	6 (86%)
**Program Design Facilitators (F)**	**5 (38%)**	**1 (14%)**
**Factors Supporting Implementation (F)**	**6 (46%)**	**1 (14%)**
**Contextual Factors (B)**	9 (69%)	4 (57%)
**Program Design Barriers (B)**	**6 (46%)**	**6 (86%)**

Note: F – Facilitator, B – Barrier; Bolded numbers and percentages are sub-themes that met the 15% difference threshold.

eTable 2.5

Maintenance Theme by participant engagement level (number of participants who identified them, percentage of engagement based on the total number of participants who identified each theme)

	Engaged (*n* = 13)	Non-Engaged (*n* = 7)
**Using Tools and Resources (F)**	**12 (92%)**	**2 (29%)**

Did Not Use Tools and Resources (B)	4 (31%)	2 (29%)

Note: F – Facilitator, B – Barrier; Bolded numbers and percentages are sub-themes that met the 15% difference threshold.

eTable 3.1

Reach Themes by Diagnosis (number of participants who identified them, percentage of participants for each diagnosis group within a theme)

	ADHD (*n* = 11)	ASD (*n* = 8)
Motivation to Participate (F)	11 (100%)	8 (100%)
**Credibility (F)**	**2 (18%)**	**3 (38%)**
Time Commitment (B)	4 (36%)	3 (38%)

Note: FASD not included here due to low representation (n=1); F – Facilitator, B – Barrier. Bolded numbers and percentages are sub-themes that met the 15% difference threshold.

eTable 3.2

Effectiveness Themes by Diagnosis (number of participants who identified them, percentage of participants for each diagnosis group within a theme)

	ADHD (*n* = 11)	ASD (*n* = 8)
**Program Design Facilitators (F)**	**6 (55%)**	**7 (88%)**
Behaviour Change Success (F)	8 (73%)	6 (75%)
**Contextual Factors (B)**	**5 (45%)**	**6 (75%)**
**Improvements to Program Design (B)**	**3 (27%)**	**4 (50%)**

Note: FASD not included here due to low representation (n=1); F – Facilitator, B – Barrier. Bolded numbers and percentages are sub-themes that met the 15% difference threshold.

eTable 3.3

Adoption Themes by Diagnosis (number of participants who identified them, percentage of participants for each diagnosis group within a theme)

	ADHD (*n* = 11)	ASD (*n* = 8)
Program Refinement (B)	**9 (82%)**	**4 (50%)**
Program Access (B)	9 (82%)	6 (75%)

Note: FASD not included here due to low representation (n=1); F – Facilitator, B – Barrier. Bolded numbers and percentages are sub-themes that met the 15% difference threshold.

eTable 3.4

Implementation Themes by Diagnosis (number of participants who identified them, percentage of participants for each diagnosis group within a theme)

	ADHD (*n* = 11)	ASD (*n* = 8)
**Level and Timing of Implementation (F)**	**10 (90%)**	**5 (63%)**
Program Design Facilitators (F)	4 (36%)	2 (25%)
Factors Supporting Implementation (F)	4 (36%)	3 (38%)
**Contextual Factors (B)**	**6 (55%)**	**6 (75%)**
**Program Design Barriers (B)**	**8 (73%)**	**4 (50%)**

Note: FASD not included here due to low representation (n=1); F – Facilitator, B – Barrier. Bolded numbers and percentages are sub-themes that met the 15% difference threshold.

eTable 3.5

Maintenance Themes by Diagnosis (number of participants who identified them, percentage of participants for each diagnosis group within a theme)

	ADHD (*n* = 11)	ASD (*n* = 8)
Using Tools and Resources (F)	11 (100%)	8 (100%)

Did Not Use Tools and Resources (B)	3 (27%)	3 (38%)

Note: FASD not included here due to low representation (n=1); F – Facilitator, B – Barrier. Bolded numbers and percentages are sub-themes that met the 15% difference threshold.

Appendix A*: BNBD-NDD* Parent Interview Script

Purpose	Question
Element 1: Reach	**For all participants, regardless of level of engagement:**<br><br>1. How did you find out about *BNBD-NDD*?<br><br>- **Prompt (if they heard of BNBD from an ad):** What aspect of the advertisement appealed to you (i.e., text, visual, message)?<br>- **Prompt (if they don't mention anything about advertisements):** Ask about different recruitment strategies (i.e., referral from healthcare provider or direct-to-consumer marketing).<br><br>2. What made you decide to participate?<br><br>3. Was there anything that made you hesitant to participate?<br><br>4. What could we do better to recruit parents of children with NDDs who have insomnia?<br><br>- **Prompt:** Provide examples of methods of recruitment (e.g., through flyer, friends, relatives, community flyers, doctor referral, phone calls, email, etc.)<br>- **Prompt:** Ask about where else we can advertise.<br>- **Prompt:** Ask about what should be included in the advertisements.
Element 2: Effectiveness	**Full, Moderate, or Minimal Engagement:**<br><br>5. Did you find *BNBD-NDD* effective?<br><br>6. What parts did you think contributed most to effectiveness?<br><br>7. Was there anything that interfered with effectiveness?<br><br>8. What could we do to make it more effective?<br><br>**No Engagement:**<br><br>9. Do you think the program would have been effective if you had been able to complete the program?
Element 3: Adoption	**For all participants, regardless of level of engagement:**<br><br>10. To sustain the intervention over time (e.g., cover costs, continue to update and further develop it) we plan to commercialize this program. Do you think parents would purchase this program? (Probe for reason)<br><br>11. What would make parents more likely to purchase the program?

	12. What would get in the way of parents purchasing the program?<br><br>13. Do you have other suggestions of how we can sustain this program over time?
Element 4: Implementation	**For all participants, regardless of level of engagement:**<br><br>14. Were you able to implement the strategies in *BNBD-NDD*?<br><br>**Full, Moderate, or Minimal Engagement:**<br><br>14.1. How frequently did you implement the strategies?<br><br>14.2. How much time did you need to implement the strategies?<br><br>15. What supported you in being able to implement the strategies?<br><br>**For all participants, regardless of level of engagement:**<br><br>16. What hindered you from being able to implement the strategies?<br><br>17. What could we do to help parents implement the strategies in *BNBD-NDD*?
Element 5: Maintenance	**Full, Moderate, or Minimal Engagement:**<br><br>18. Do you think you will continue to use the strategies you learned in *BNBD-NDD*?<br><br>19. After you completed the program did you ever refer back to the program by going online and reviewing *BNBD-NDD*?<br><br>**For all participants, regardless of level of engagement:**<br><br>20. How long do you think parents would want/need access to the program? Why?<br><br>21. What could we do to help parents continue to implement the strategies in this program over the longer term?
	22. At this point, are there any other barriers or facilitators to participating in Better Nights, Better Days program that we have not discussed that you would like to add?<br><br>23. Do you have any final comments or questions?
Wrap-Up	

Thank you very much for taking the time to participate in this interview with us today. The feedback you gave will help us improve the *BNBD- NDD* program, which can help more parents of children with neurodevelopment disorders to sleep better!
